# Quinine Not Good in Hemorrhagic Malarial Fever

**Published:** 1887-05

**Authors:** 


### QUININE NOT GOOD IN HEMORRHAGIC MALARIAL FEVER.

Dr. E. H. M. Parrham, of Fordyce, Ark., writes :

"I would dissuade our professional brethren from the use of quinine in hemorrhagic malarial fever. I have just treated an aggravated case successfully which demonstrated to my mind that quinine has caused the death of many, and benefitted none."

We hope the Doctor will report his case in detail at an early day. We thank him for the following compliment paid to our journal :

"I am glad to say that I believe the SOUTHERN MEDICAL RECORD the best and most practical journal published in the United States."

